# The genome sequence of the Tawny Mining Bee, *Andrena fulva *(Müller, 1766)

**DOI:** 10.12688/wellcomeopenres.19510.1

**Published:** 2023-06-21

**Authors:** Liam M. Crowley, John F. Mulley, University of Oxford and Wytham Woods Genome Acquisition Lab, Darwin Tree of Life Barcoding collective, Wellcome Sanger Institute Tree of Life programme, Wellcome Sanger Institute Scientific Operations: DNA Pipelines collective, Tree of Life Core Informatics collective, Darwin Tree of Life Consortium

**Affiliations:** 1 University of Oxford, Oxford, England, UK; 2 School of Natural Sciences, Bangor University, Bangor, Wales, UK

**Keywords:** Andrena fulva, Tawny Mining Bee, genome sequence, chromosomal, Hymenoptera

## Abstract

We present a genome assembly from an individual female *Andrena fulva* (the Tawny Mining Bee; Arthropoda; Insecta; Hymenoptera; Andrenidae). The genome sequence is 461.7 megabases in span. Most of the assembly is scaffolded into 7 chromosomal pseudomolecules. The mitochondrial genome has also been assembled and is 14.7 kilobases in length. Gene annotation of this assembly on Ensembl identified 12,011 protein coding genes.

## Species taxonomy

Eukaryota; Metazoa; Eumetazoa; Arthropoda; Mandibulata; Pancrustacea; Hexapoda; Insecta; Dicondylia; Pterygota; Neoptera; Endopterygota; Hymenoptera; Apocrita; Aculeata; Apoidea; Anthophila; Andrenidae; Andreninae; *Andrena*; *Andrena*; *Andrena fulva* (Müller, 1766) (NCBI:txid1411667).

## Background

The Tawny Mining Bee, *Andrena fulva,* is a common and widespread mining bee in the UK. Females are large (10 mm wing length) and are one of the most distinctive within the *Andrena* genus, with long red hairs dorsally across the thorax and abdomen and black-haired head and legs. The smaller males (8 mm wing length) are less striking, with generally duller red-brown hairs on the body, although they may be recognised by the white hairs on the clypeus and long mandibles with a distinct tooth at the base ( Paxton, 1991).

*Andrena fulva* is distributed throughout western Europe, east to the Balkans and north to southern Scandinavia. In the UK, it is locally common across England and Wales and may be found north to southern Scotland. It occurs in a range of habitats, in particular open grasslands, pastures and parks and gardens. It is broadly polylectic, visiting a range of flowers including beech ( *Fagus sylvatica*), blackthorn ( *Prunus spinosa*), buttercup ( *Ranunculus* sp.), garlic mustard ( *Alliaria petiolata*), current ( *Ribes* sp.), hawthorn ( *Crataegus monogyna*), maple ( *Acer* sp.), oak ( *Quercus* sp.), plum ( *Prunus domestica*) and sallow ( *Salix* sp.) ( Chambers, 1968).

The species is univoltine, with a flight period from March to May. Males generally emerge first, before the females which mate then build and provision two or three nests ( O’Toole & Raw, 1991). In accordance with the wider genus, nests are constructed underground. Nesting typically occurs on level, loose sandy soil with short, sparge vegetation ( Maher *et al.*, 2019). Females excavate a main central vertical tunnel 20 – 40 cm deep, from which arise 4 or 5 radial cells ( O’Toole & Raw, 1991). The excavated soil often forms a volcano-like tumulus which may be conspicuous in short lawns. *Andrena fulva* frequently forms dense nesting aggregations, that may persist in the same location for several years. Such aggregations are likely to be in response to resource distribution (e.g. near floral resources or suitable nesting substrate), although it had also been suggested that they may confer a degree of natural enemy escape ( Hamilton, 1971; Rosenheim, 1990). Such aggregated nesting may represent an early step in the evolution of true sociality.

This *Andrena fulva* genome assembly will prove invaluable in the development of genetic markers to investigate relatedness among aggregated individuals. While a large number of females in the related *Andrena jacobi* commonly mate with male nest-mates pre-emergence ( Paxton & Tengö, 1996; Paxton *et al.*, 1996), this seems not to be the case in *A. fulva*, in which males tend to emerge before females ( Paxton, 1991). 

### Genome sequence report

**Figure 1.**
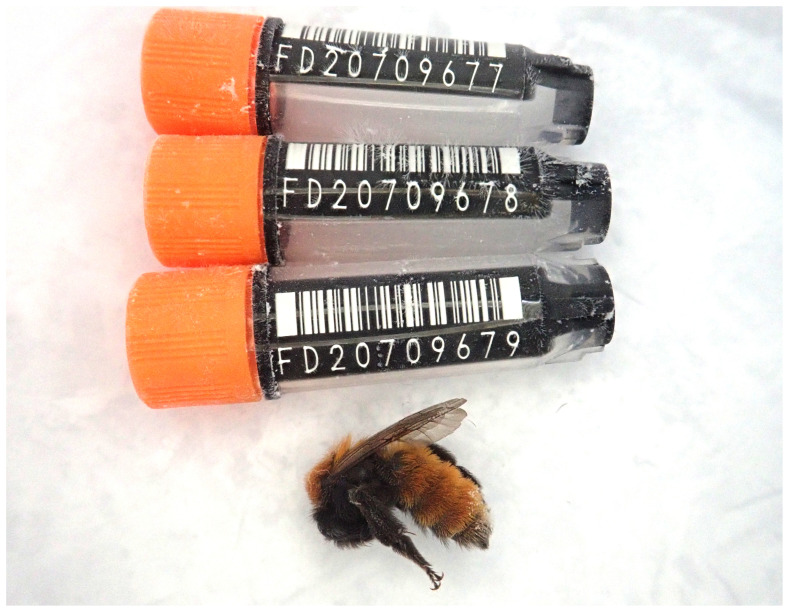
Photograph of the *Andrena fulva* (iyAndFulv1) specimen used for genome sequencing.

The genome was sequenced from one female *Andrena fulva* ( Figure 1) collected from Wytham Farm, Oxfordshire, UK (51.79, –1.32). A total of 46-fold coverage in Pacific Biosciences single-molecule HiFi long reads was generated. Primary assembly contigs were scaffolded with chromosome conformation Hi-C data. Manual assembly curation corrected 14 missing joins or mis-joins and removed one haplotypic duplication, reducing the assembly length by 0.22% and the scaffold number by 3.94%, and increasing the scaffold N50 by 113.54%.

**Figure 2.**
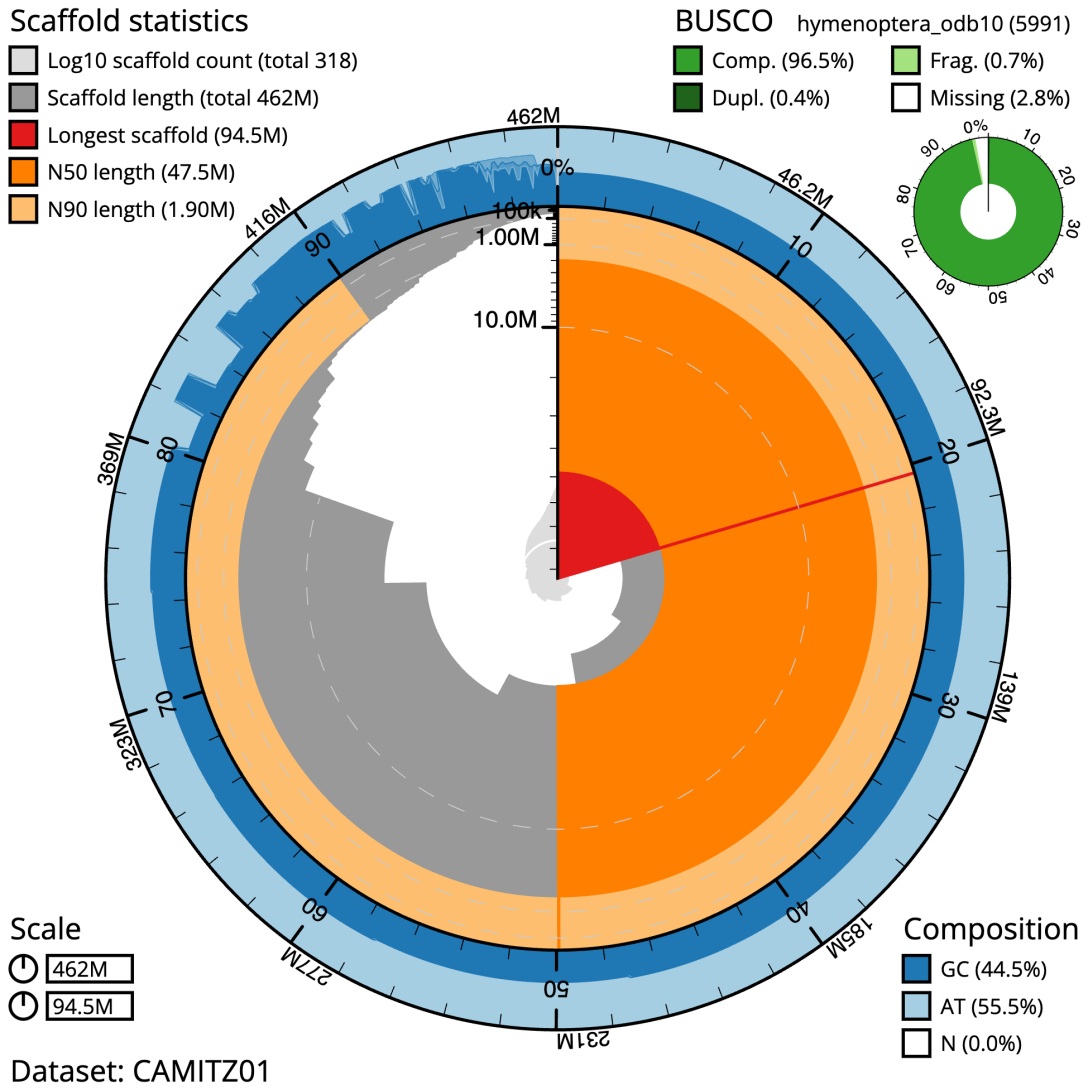
Genome assembly of *Andrena fulva*, iyAndFulv1.1: metrics. The BlobToolKit Snailplot shows N50 metrics and BUSCO gene completeness. The main plot is divided into 1,000 size-ordered bins around the circumference with each bin representing 0.1% of the 461,725,953 bp assembly. The distribution of scaffold lengths is shown in dark grey with the plot radius scaled to the longest scaffold present in the assembly (94,538,841 bp, shown in red). Orange and pale-orange arcs show the N50 and N90 scaffold lengths (47,475,851 and 1,904,000 bp), respectively. The pale grey spiral shows the cumulative scaffold count on a log scale with white scale lines showing successive orders of magnitude. The blue and pale-blue area around the outside of the plot shows the distribution of GC, AT and N percentages in the same bins as the inner plot. A summary of complete, fragmented, duplicated and missing BUSCO genes in the hymenoptera_odb10 set is shown in the top right. An interactive version of this figure is available at https://blobtoolkit.genomehubs.org/view/iyAndFulv1.1/dataset/CAMITZ01/snail.

The final assembly has a total length of 461.7 Mb in 317 sequence scaffolds with a scaffold N50 of 47.5 Mb ( Table 1). Most (80.24%) of the assembly sequence was assigned to 7 chromosomal-level scaffolds. Chromosome-scale scaffolds confirmed by the Hi-C data are named in order of size ( Figure 2– Figure 5; Table 2). While not fully phased, the assembly deposited is of one haplotype. Contigs corresponding to the second haplotype have also been deposited. The mitochondrial genome was also assembled and can be found as a contig within the multifasta file of the genome submission.

**Table 1.** Genome data for *Andrena fulva*, iyAndFulv1.1.

Project accession data		
Assembly identifier	iyAndFulv1.1	
Species	*Andrena fulva*	
Specimen	iyAndFulv1	
NCBI taxonomy ID	1411667	
BioProject	PRJEB54055	
BioSample ID	SAMEA10166729	
Isolate information	iyAndFulv1, female: thorax (genome sequencing), abdomen (RNA sequencing), head (Hi-C scaffolding)	
Assembly metrics *		*Benchmark*
Consensus quality (QV)	62.4	*≥ 50*
*k*-mer completeness	100%	*≥ 95%*
BUSCO **	C:96.5%[S:96.1%,D:0.4%], F:0.7%,M:2.8%,n:5,991	*C ≥ 95%*
Percentage of assembly mapped to chromosomes	80.24%	*≥ 95%*
Sex chromosomes	-	*localised homologous pairs*
Organelles	Mitochondrial genome assembled	*complete single alleles*
Raw data accessions		
PacificBiosciences SEQUEL II	ERR9924614	
Hi-C Illumina	ERR9930689	
PolyA RNA-Seq Illumina	ERR10890692	
Genome assembly		
Assembly accession	GCA_946251845.1	
*Accession of alternate haplotype*	GCA_946251835.1	
Span (Mb)	461.7	
Number of contigs	358	
Contig N50 length (Mb)	12.5	
Number of scaffolds	317	
Scaffold N50 length (Mb)	47.5	
Longest scaffold (Mb)	94.5	
Genome annotation		
Number of protein-coding genes	12,011	
Number of non-coding genes	6,025	
Number of gene transcripts	28,635	

* Assembly metric benchmarks are adapted from column VGP-2020 of “Table 1: Proposed standards and metrics for defining genome assembly quality” from ( Rhie *et al.*, 2021). ** BUSCO scores based on the hymenoptera_odb10 BUSCO set using v5.3.2. C = complete [S = single copy, D = duplicated], F = fragmented, M = missing, n = number of orthologues in comparison. A full set of BUSCO scores is available at https://blobtoolkit.genomehubs.org/view/iyAndFulv1.1/dataset/CAMITZ01/busco.

**Figure 3.**
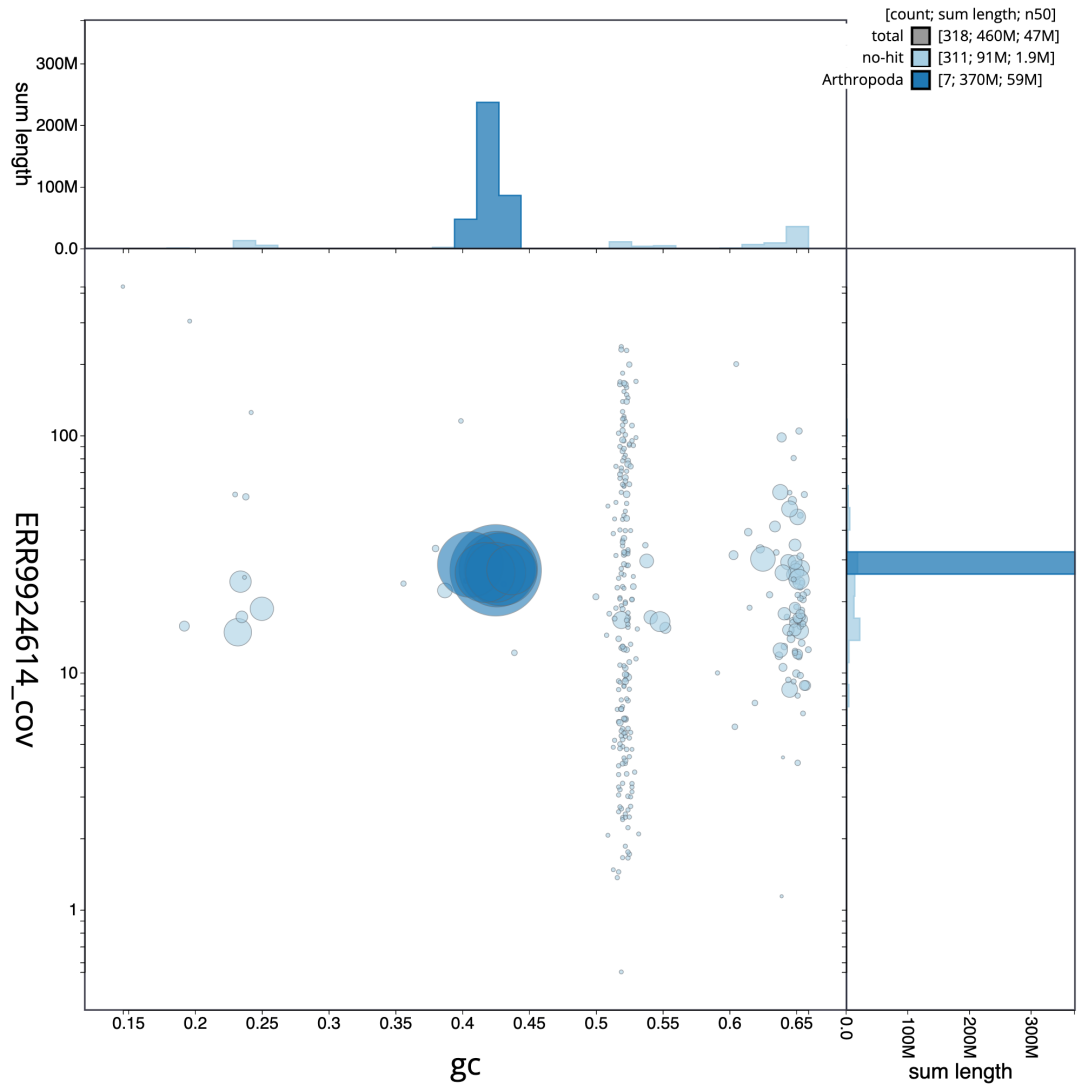
Genome assembly of *Andrena fulva*, iyAndFulv1.1: BlobToolKit GC-coverage plot. Scaffolds are coloured by phylum. Circles are sized in proportion to scaffold length. Histograms show the distribution of scaffold length sum along each axis. An interactive version of this figure is available at https://blobtoolkit.genomehubs.org/view/iyAndFulv1.1/dataset/CAMITZ01/blob.

**Figure 4.**
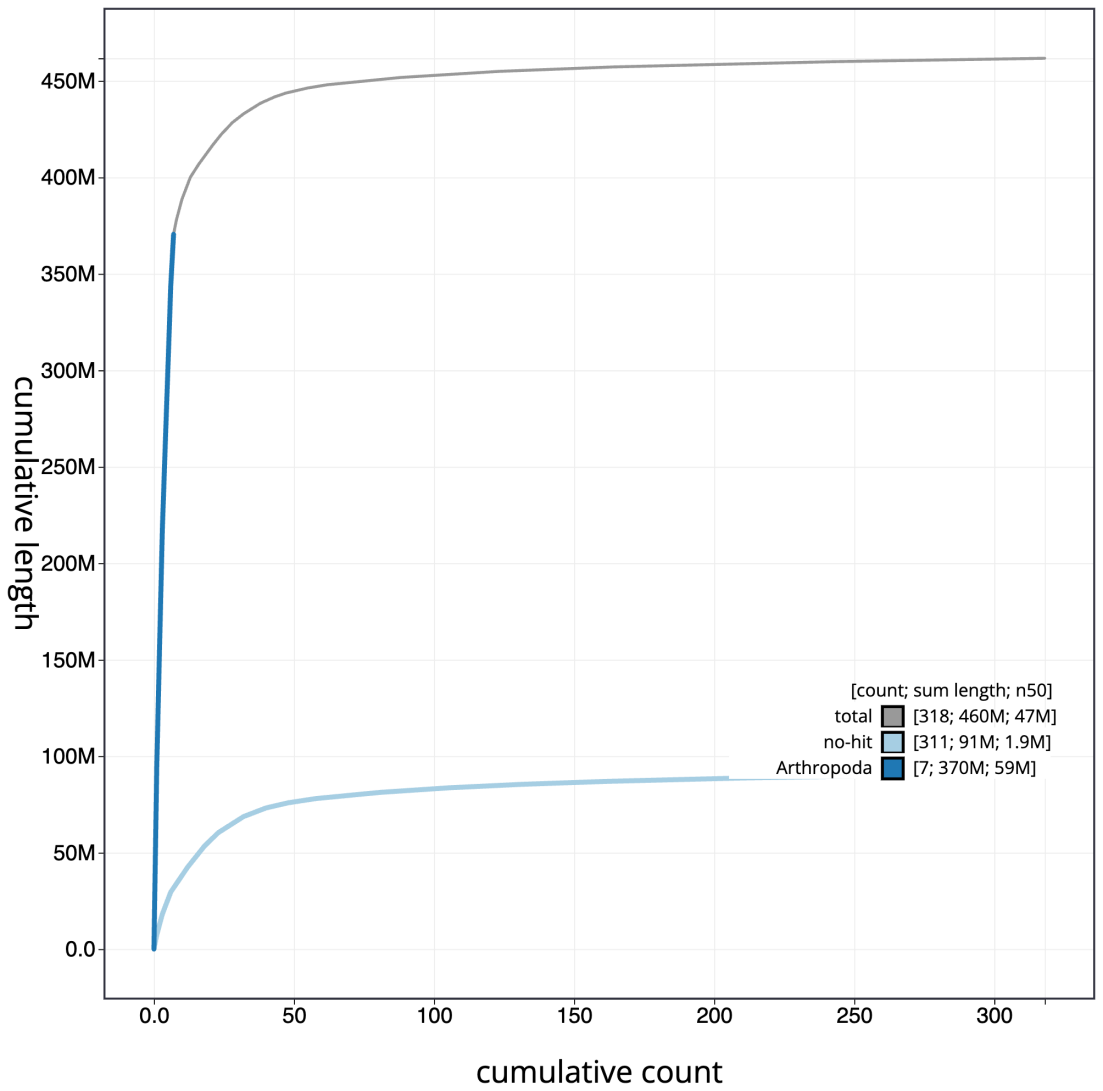
Genome assembly of *Andrena fulva*, iyAndFulv1.1: BlobToolKit cumulative sequence plot. The grey line shows cumulative length for all scaffolds. Coloured lines show cumulative lengths of scaffolds assigned to each phylum using the buscogenes taxrule. An interactive version of this figure is available at https://blobtoolkit.genomehubs.org/view/iyAndFulv1.1/dataset/CAMITZ01/cumulative.

**Figure 5.**
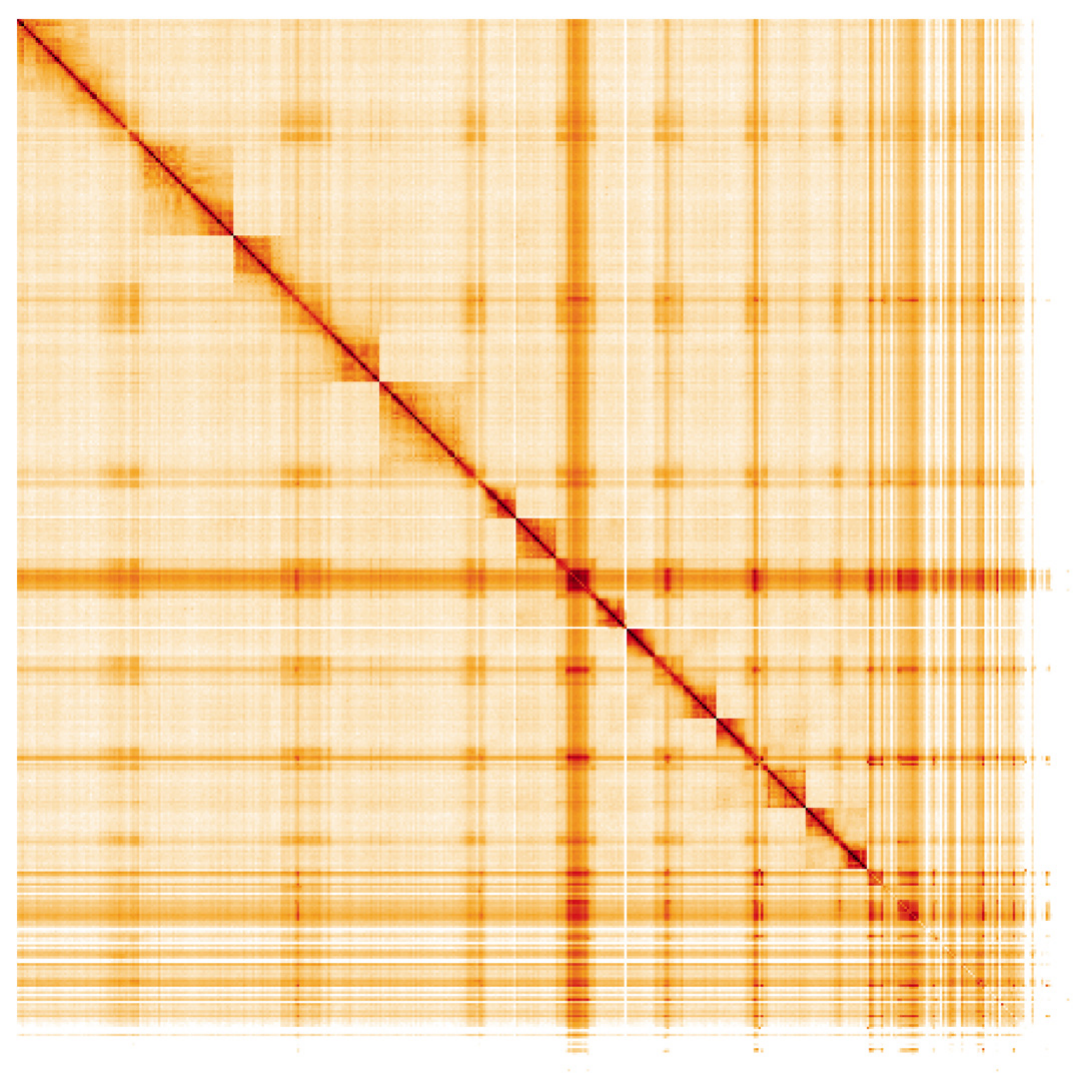
Genome assembly of *Andrena fulva*, iyAndFulv1.1: Hi-C contact map of the iyAndFulv1.1 assembly, visualised using HiGlass. Chromosomes are shown in order of size from left to right and top to bottom. An interactive version of this figure may be viewed at https://genome-note-higlass.tol.sanger.ac.uk/l/?d=PTK-eNDiQ7eRqSCzUJOPQw.

**Table 2.** Chromosomal pseudomolecules in the genome assembly of *Andrena fulva*, iyAndFulv1.

INSDC accession	Name	Length (Mb)	GC%
OX276327.1	1	94.54	42.5
OX276328.1	2	64.01	42.5
OX276329.1	3	59.22	43
OX276330.1	4	47.48	40.5
OX276331.1	5	39.29	42.5
OX276332.1	6	39.23	41.5
OX276333.1	7	26.74	43.5
OX276334.1	MT	0.01	23.5

The estimated Quality Value (QV) of the final assembly is 62.4 with *k*-mer completeness of 100%, and the assembly has a BUSCO v5.3.2 completeness of 96.5% (single = 96.1%, duplicated = 0.4%), using the hymenoptera_odb10 reference set ( *n* = 5,991).

Metadata for specimens, spectral estimates, sequencing runs, contaminants and pre-curation assembly statistics can be found at https://links.tol.sanger.ac.uk/species/1411667.

### Genome annotation report

The *Andrena fulva* genome assembly (GCA_946251845.1) was annotated using the Ensembl rapid annotation pipeline ( Table 1; https://rapid.ensembl.org/Andrena_fulva_GCA_946251845.1/Info/Index). The resulting annotation includes 28,635 transcribed mRNAs from 12,011 protein-coding and 6,025 non-coding genes.

## Methods

### Sample acquisition and nucleic acid extraction

A female *Andrena fulva* (specimen number Ox001244, individual iyAndFulv1) was collected from Wytham Farm, Oxfordshire (biological vice-county Berkshire), UK (latitude 51.79, longitude –1.32) on 2021-04-19. The specimen was taken from Agricultural land by Liam Crowley (University of Oxford) by netting. The specimen was identified by Liam Crowley and snap-frozen on dry ice.

DNA was extracted at the Tree of Life laboratory, Wellcome Sanger Institute (WSI). The iyAndFulv1 sample was weighed and dissected on dry ice with tissue set aside for Hi-C sequencing. Thorax tissue was disrupted using a Nippi Powermasher fitted with a BioMasher pestle. High molecular weight (HMW) DNA was extracted using the Qiagen MagAttract HMW DNA extraction kit. HMW DNA was sheared into an average fragment size of 12–20 kb in a Megaruptor 3 system with speed setting 30. Sheared DNA was purified by solid-phase reversible immobilisation using AMPure PB beads with a 1.8× ratio of beads to sample to remove the shorter fragments and concentrate the DNA sample. The concentration of the sheared and purified DNA was assessed using a Nanodrop spectrophotometer and Qubit Fluorometer and Qubit dsDNA High Sensitivity Assay kit. Fragment size distribution was evaluated by running the sample on the FemtoPulse system.

RNA was extracted from abdomen tissue of iyAndFulv1 in the Tree of Life Laboratory at the WSI using TRIzol, according to the manufacturer’s instructions. RNA was then eluted in 50 μl RNAse-free water and its concentration assessed using a Nanodrop spectrophotometer and Qubit Fluorometer using the Qubit RNA Broad-Range (BR) Assay kit. Analysis of the integrity of the RNA was done using Agilent RNA 6000 Pico Kit and Eukaryotic Total RNA assay.

### Sequencing

Pacific Biosciences HiFi circular consensus DNA sequencing libraries were constructed according to the manufacturers’ instructions. Poly(A) RNA-Seq libraries were constructed using the NEB Ultra II RNA Library Prep kit. DNA and RNA sequencing was performed by the Scientific Operations core at the WSI on Pacific Biosciences SEQUEL II (HiFi) and Illumina NovaSeq 6000 (RNA-Seq) instruments. Hi-C data were also generated from head tissue of iyAndFulv1 using the Arimav2 kit and sequenced on the Illumina NovaSeq 6000 instrument.

### Genome assembly, curation and evaluation

Assembly was carried out with Hifiasm ( Cheng *et al.*, 2021) and haplotypic duplication was identified and removed with purge_dups ( Guan *et al.*, 2020). The assembly was then scaffolded with Hi-C data ( Rao *et al.*, 2014) using YaHS ( Zhou *et al.*, 2023). The assembly was checked for contamination as described previously ( Howe *et al.*, 2021). Manual curation was performed using HiGlass ( Kerpedjiev *et al.*, 2018) and Pretext ( Harry, 2022). The mitochondrial genome was assembled using MitoHiFi ( Uliano-Silva *et al.*, 2022), which runs MitoFinder ( Allio *et al.*, 2020) or MITOS ( Bernt *et al.*, 2013) and uses these annotations to select the final mitochondrial contig and to ensure the general quality of the sequence.

A Hi-C map for the final assembly was produced using bwa-mem2 ( Vasimuddin *et al.*, 2019) in the Cooler file format ( Abdennur & Mirny, 2020). To assess the assembly metrics, the *k*-mer completeness and QV consensus quality values were calculated in Merqury ( Rhie *et al.*, 2020). This work was done using Nextflow ( Di Tommaso *et al.*, 2017) DSL2 pipelines “sanger-tol/readmapping” ( Surana *et al.*, 2023a) and “sanger-tol/genomenote” ( Surana *et al.*, 2023b). The genome was analysed within the BlobToolKit environment ( Challis *et al.*, 2020) and BUSCO scores ( Manni *et al.*, 2021; Simão *et al.*, 2015) were calculated.

Table 3 contains a list of relevant software tool versions and sources.

**Table 3.** Software tools: versions and sources.

Software tool	Version	Source
BlobToolKit	4.0.7	https://github.com/blobtoolkit/blobtoolkit
BUSCO	5.3.2	https://gitlab.com/ezlab/busco
Hifiasm	0.16.1-r375	https://github.com/chhylp123/hifiasm
HiGlass	1.11.6	https://github.com/higlass/higlass
Merqury	MerquryFK	https://github.com/thegenemyers/MERQURY.FK
MitoHiFi	2	https://github.com/marcelauliano/MitoHiFi
PretextView	0.2	https://github.com/wtsi-hpag/PretextView
purge_dups	1.2.3	https://github.com/dfguan/purge_dups
sanger-tol/genomenote	v1.0	https://github.com/sanger-tol/genomenote
sanger-tol/readmapping	1.1.0	https://github.com/sanger-tol/readmapping/tree/1.1.0
YaHS	yahs-1.1.91eebc2	https://github.com/c-zhou/yahs

### Genome annotation

The Ensembl gene annotation system ( Aken *et al.*, 2016) was used to generate annotation for the *Andrena fulva* assembly (GCA_946251845.1). Annotation was created primarily through alignment of transcriptomic data to the genome, with gap filling via protein-to-genome alignments of a select set of proteins from UniProt ( UniProt Consortium, 2019).

### Wellcome Sanger Institute – Legal and Governance

The materials that have contributed to this genome note have been supplied by a Darwin Tree of Life Partner.

The submission of materials by a Darwin Tree of Life Partner is subject to the **‘Darwin Tree of Life Project Sampling Code of Practice’**, which can be found in full on the Darwin Tree of Life website here. By agreeing with and signing up to the Sampling Code of Practice, the Darwin Tree of Life Partner agrees they will meet the legal and ethical requirements and standards set out within this document in respect of all samples acquired for, and supplied to, the Darwin Tree of Life Project.

Further, the Wellcome Sanger Institute employs a process whereby due diligence is carried out proportionate to the nature of the materials themselves, and the circumstances under which they have been/are to be collected and provided for use. The purpose of this is to address and mitigate any potential legal and/or ethical implications of receipt and use of the materials as part of the research project, and to ensure that in doing so we align with best practice wherever possible.

The overarching areas of consideration are:

- Ethical review of provenance and sourcing of the material
- Legality of collection, transfer and use (national and international)

Each transfer of samples is further undertaken according to a Research Collaboration Agreement or Material Transfer Agreement entered into by the Darwin Tree of Life Partner, Genome Research Limited (operating as the Wellcome Sanger Institute), and in some circumstances other Darwin Tree of Life collaborators.

## Data Availability

European Nucleotide Archive: *Andrena fulva* (tawny mining bee). Accession number PRJEB54055; https://identifiers.org/ena.embl/PRJEB54055. ( Wellcome Sanger Institute, 2022) The genome sequence is released openly for reuse. The *Andrena fulva* genome sequencing initiative is part of the Darwin Tree of Life (DToL) project. All raw sequence data and the assembly have been deposited in INSDC databases. Raw data and assembly accession identifiers are reported in Table 1.

## Author information

Members of the University of Oxford and Wytham Woods Genome Acquisition Lab are listed here: https://doi.org/10.5281/zenodo.4789928.

Members of the Darwin Tree of Life Barcoding collective are listed here: https://doi.org/10.5281/zenodo.4893703.

Members of the Wellcome Sanger Institute Tree of Life programme are listed here: https://doi.org/10.5281/zenodo.4783585.

Members of Wellcome Sanger Institute Scientific Operations: DNA Pipelines collective are listed here: https://doi.org/10.5281/zenodo.4790455.

Members of the Tree of Life Core Informatics collective are listed here: https://doi.org/10.5281/zenodo.5013541.

Members of the Darwin Tree of Life Consortium are listed here: https://doi.org/10.5281/zenodo.4783558.
