# Supplementary material for: The genome sequence of the Tawny Mining Bee, Andrena fulva (Müller, 1766)
Source: Wellcome Open Res. 2023 Jun 21;8:258. [Version 1] doi: 10.12688/wellcomeopenres.19510.1 (PMC11480741; doi:10.12688/wellcomeopenres.19510.1)
Supplement: Peer review history

# Reviewer report: Reviewer response for version 1

**DOI:** 10.21956/wellcomeopenres.21612.r101818

**Published:** 2024-10-15

**Authors:** Željko Tomanović

**Affiliations:** 1 Serbia University of Belgrade, Belgrade, Serbia

The genome of The Tawny Mining Bee, *Andrena fulva* (Muller, 1766), has been sequenced. The authors have provided excellent background information on the biology and ecology of this interesting solitary bee species. I recommend authors for using mesosoma instead of thorax and metasoma instead of abdomen (usually within apocritan Hymenoptera), but this is not mandatory. The genome is described and illustrated in great detail. The methodology used is appropriate and explained in detail. The paper is very well written and I recommend it for indexing.

Are sufficient details of methods and materials provided to allow replication by others?

Yes

Is the rationale for creating the dataset(s) clearly described?

Yes

Are the datasets clearly presented in a useable and accessible format?

Yes

Are the protocols appropriate and is the work technically sound?

Yes

Reviewer Expertise:

Biodiversity, ecology and phylogenetics

I confirm that I have read this submission and believe that I have an appropriate level of expertise to confirm that it is of an acceptable scientific standard.

# Reviewer report: Reviewer response for version 1

**DOI:** 10.21956/wellcomeopenres.21612.r84841

**Published:** 2024-06-11

**Authors:** Shannon Hedtke

**Affiliations:** 1 La Trobe University, Bundoora, Victoria, Australia

Crowley et al. sequence the genome of the Tawny Mining Bee, *Andrena fulva*, a charismatic and polylectic bee which can form nesting aggregations. Identifying key steps (both behavioural and genomic) in the evolution of eusociality remains a major research question. This work thus contributes towards exploration of social behavior, including identifying whether aggregations contain closely related individuals.

 The manuscript is very clear and well-written. To ensure reproducibility, I would recommend adding more detail about how missing joins/mis-joins/haplotypic duplications were identified during manual curation.

 The comma in the following sentence should also be removed: " *Andrena fulva* frequently forms dense nesting aggregations, that may persist in the same location for several years."

Are sufficient details of methods and materials provided to allow replication by others?

Partly

Is the rationale for creating the dataset(s) clearly described?

Yes

Are the datasets clearly presented in a useable and accessible format?

Yes

Are the protocols appropriate and is the work technically sound?

Yes

Reviewer Expertise:

My expertise is in genomics, entomology, parasitology, and population and phylogenetics.

I confirm that I have read this submission and believe that I have an appropriate level of expertise to confirm that it is of an acceptable scientific standard.
